# Emerging Role of the Nucleolar Stress Response in Autophagy

**DOI:** 10.3389/fncel.2019.00156

**Published:** 2019-04-30

**Authors:** Astrid S. Pfister

**Affiliations:** Institute of Biochemistry and Molecular Biology, Faculty of Medicine, Ulm University, Ulm, Germany

**Keywords:** ribosome biogenesis, rRNA processing, nucleoli, nucleolar stress, autophagy, neuron, mitochondria, aggregates

## Abstract

Autophagy represents a conserved self-digestion program, which allows regulated degradation of cellular material. Autophagy is activated by cellular stress, serum starvation and nutrient deprivation. Several autophagic pathways have been uncovered, which either non-selectively or selectively target the cellular cargo for lysosomal degradation. Autophagy engages the coordinated action of various key regulators involved in the steps of autophagosome formation, cargo targeting and lysosomal fusion. While non-selective (macro)autophagy is required for removal of bulk material or recycling of nutrients, selective autophagy mediates specific targeting of damaged organelles or protein aggregates. By proper action of the autophagic machinery, cellular homeostasis is maintained. In contrast, failure of this fundamental process is accompanied by severe pathophysiological conditions. Hallmarks of neuropathological disorders are for instance accumulated, mis-folded protein aggregates and damaged mitochondria. The nucleolus has been recognized as central hub in the cellular stress response. It represents a sub-nuclear organelle essential for ribosome biogenesis and also functions as stress sensor by mediating cell cycle arrest or apoptosis. Thus, proper nucleolar function is mandatory for cell growth and survival. Here, I highlight the emerging role of nucleolar factors in the regulation of autophagy. Moreover, I discuss the nucleolar stress response as a novel signaling pathway in the context of autophagy, health and disease.

## Introduction

Various high quality reviews are available on principles of ribosome biogenesis, nucleolar stress, apoptosis and autophagy, respectively. Given their essential role, it is well accepted that a mis-regulation of each is tightly linked to pathogenic conditions (Levine and Kroemer, 2008; Boulon et al., 2010; Freed et al., 2010; Ghavami et al., 2014; Schneider and Cuervo, 2014). In this review, the emerging connection of nucleolar stress to autophagic processes serves as a basis to discuss novel concepts and cure of diseases connected to nucleolar stress.

### The Nucleolus as a Stress Sensor

#### The Nucleolus: Place for Ribosome Biogenesis

Nucleoli represent membrane-free, sub-nuclear compartments, where transcription and processing of rRNA takes place. Nucleoli can be considered as an assembly platform. They host several hundreds of essential rRNA binding and processing factors, which are involved in the highly complex process of ribosome biogenesis. Nucleoli form around repetitive rDNA clusters in a dynamic and cell cycle-dependent manner during G1 phase (Potmesil and Goldfeder, 1977; Mangan et al., 2017).

The rDNA clusters are transcribed into their respective large precursor rRNA by RNA polymerase I (RNA pol I); RNA polymerases II and III are as well essential for ribosome biogenesis, by driving the expression of ribosomal proteins (RNA pol II) and 5S rRNA (RNA pol III) (Eichler and Craig, 1994; Fatica and Tollervey, 2002). The complex mechanism of pre-rRNA processing involves the action of a multitude of ribosome biogenesis factors. These are assembled in pre-ribosomal complexes involved in cleavage and chemical modification of the maturating transcript (Fatica and Tollervey, 2002; Granneman and Baserga, 2004; Mullineux and Lafontaine, 2012).

The nucleolar size correlates with the rRNA transcription, cell growth and the metabolic rate of a cell (Boulon et al., 2010). Importantly, nucleolar size and function is changed during aging (Tiku et al., 2016; Buchwalter and Hetzer, 2017; Zlotorynski, 2017). Thus, the nucleolus emerges as critical regulator of cellular aging (Tiku and Antebi, 2018).

Large amounts of ribosomes are especially needed in highly proliferating cells, such as during embryonic development or cancer (Montanaro et al., 2008). Therefore, a lack of functional ribosomes impairs cellular growth and survival and is incompatible with life.

#### The Nucleolar Stress Response

Nucleoli are highly dynamic structures, closely connected to growth and survival (Mangan et al., 2017). The nucleolus is being recognized as a key hub in the cellular stress response by sensing and reacting to various stimuli.

**FIGURE 1:**
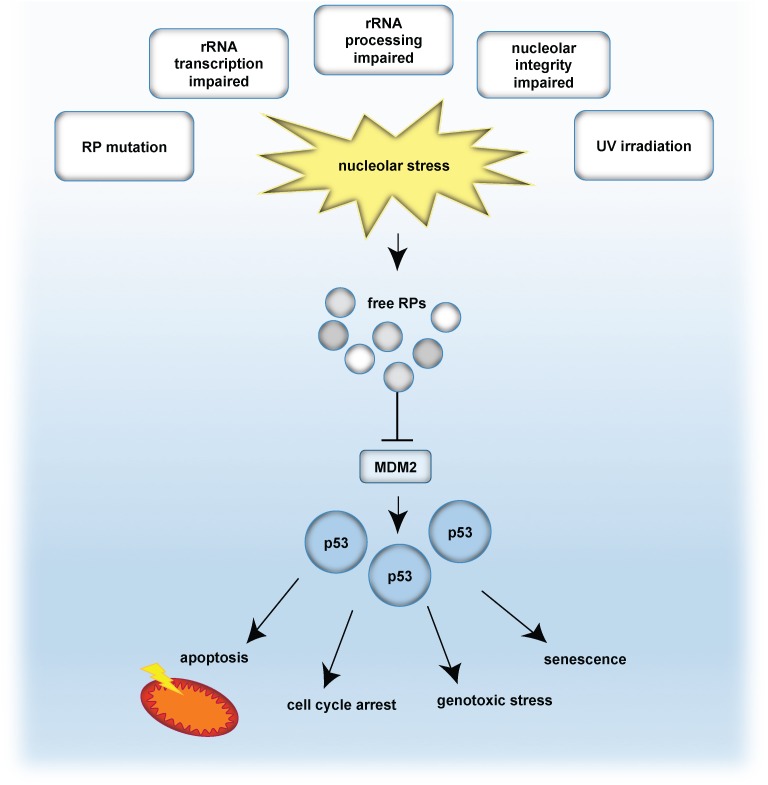
The classical p53-dependent nucleolar stress response pathway. Nucleolar stress is caused by e.g., mutation of ribosomal proteins (RP), impaired transcription of rDNA into rRNA, abrogated rRNA processing, disrupted nucleolar integrity as well as by genotoxic stressors, such as UV irradiation. As a consequence, RPs are released and bind and inhibit the E3-ubiquitin ligase MDM2. In turn, p53 is no longer degraded in the proteasome and is stabilized. Given the p53 accumulation, p53-mediated effects are propagated, such as cell cycle arrest, senescence, apoptosis or genotoxic stress. Note that also p53-independent routes exist, which are not indicated in this scheme.

Perturbation of the nucleolar structure and/or function ultimately impairs ribosome biogenesis and triggers the so-called nucleolar stress response (Boulon et al., 2010). A key mechanism involves the release of ribosomal proteins (RPs) from the nucleoli into the nucleoplasm. As a consequence of nucleoplasmic RP accumulation, the E3-ubiquitin ligase MDM2 is inhibited (Dai et al., 2004). MDM2 keeps the levels of the tumor suppressor protein p53 low by earmarking p53 for proteasomal degradation. Upon nucleolar stress, RPs are released and inhibit MDM2, which results in p53 accumulation. Finally, stabilized p53 induces cell cycle arrest and/or apoptosis (Pestov et al., 2001; Rubbi and Milner, 2003; Yuan et al., 2005; Fumagalli et al., 2012). The nucleolar stress response is further connected to the induction of senescence and DNA damage, by commonly engaging the classical p53 pathway (Rubbi and Milner, 2003; Lindstrom et al., 2018). A simplified model of the classical p53 nucleolar stress response is given in Figure 1.

More recently, novel pathways have been added to the increasing list, which demonstrate that nucleolar stress can also be propagated in the absence of functional p53 (Holmberg Olausson et al., 2012; James et al., 2014; Lindstrom et al., 2018).

In summary, nucleolar integrity reflects a general prerequisite for normal cellular function. Given that many tumor types are characterized by inactivation of p53, p53-independent pathways open novel avenues toward more customized anti-cancer therapies (Burger and Eick, 2013).

### Autophagy Pathways

#### Macroautophagy

(Macro)autophagy is essential for cellular homeostasis by mediating destruction and recycling of bulk cytoplasmic material, defective organelles or proteins via lysosomal degradation (Mizushima, 2007; Marx, 2015). A mis-regulation of autophagy is tightly linked to the formation of diverse pathological conditions (Levine and Kroemer, 2008; Jiang and Mizushima, 2014; Schneider and Cuervo, 2014).

Autophagy can be induced by various cellular stresses, such as lack of nutrients, low energy or oxidative stress. As an upstream signaling pathway the conserved mTOR pathway plays an influential role in the regulation of autophagy (see section “mTOR Signaling Couples Autophagy and Ribosome Biogenesis”) (Pattingre et al., 2008).

A central structure implicated in the process of (macro)autophagy is the double-membranous autophagosome, which mediates cellular cargo sequestration. Autophagy-related proteins (ATGs) govern autophagosome formation at different levels. Beclin1, the mammalian homologue of yeast Atg6, is mandatory for the initial steps of autophagosome formation (Pattingre et al., 2008). Originally, Beclin1 has been identified as an interaction partner of the anti-apoptotic factor Bcl-2 (B cell lymphoma 2) (Liang et al., 1998). Beclin1 is part of phosphoinoside 3 kinase (PI3K) complexes and functions in diverse membrane trafficking processes (Levine et al., 2015). Furthermore, Beclin1 interacts with kinases, de-/ubiquitinating enzymes and multiple other factors, among them p53 (Levine et al., 2015). Certain ATGs are necessary for the engulfment of cargo destined for lysosomal degradation in the autolysosome. Members of the Atg8 protein family are directly conjugated to the autophagosomal membrane by phosphaditylinositol lipidation. Hence, Atg8/LC3 (light chain 3) represents a key marker of autophagosomes. Atg8/LC3s are essential for autophagosome maturation and cargo sequestration. They can be sub-divided into LC3 and GABARAP proteins and fulfill critical tasks (Nguyen et al., 2016).

Note that intracellular Atg8/LC3-positive autophagosome accumulation is central for autophagy flux induction as well as inhibition. Opposing mechanisms result in the accumulation of autophagosomes, thereby requiring careful interpretation of experimental results. An increased rate of autophagosome formation (increase of autophagic flux), as well as decreased autophagosome clearance in the lysosome (impaired autophagic flux), resembles autophagosome accumulation under basal conditions. Thus, care has to be taken when interpreting results on “active” or “inhibited” autophagy. Meanwhile, several excellent reviews are available, which help to unravel these issues (Mizushima and Yoshimori, 2007; Mizushima et al., 2010; Klionsky, 2011; Gottlieb et al., 2015; Klionsky et al., 2016). So-called autophagic flux studies have become detrimental for understanding mechanisms of autophagy. Experts agree on combining various independent methods to allow solid data interpretation (Klionsky et al., 2016).

In general, autophagy is noticed as a protective mechanism by lowering the cellular stress.

Apoptosis and autophagy are both stimulated by similar stressors. However, they can be seen as opposing signaling events. Whereas autophagy acts in an anti-apoptotic manner and precedes apoptosis (Boya et al., 2005), apoptosis induction can block autophagy for instance by removing pro-autophagy proteins by caspases. However, this can even generate pro-apoptotic fragments of ATG autophagy regulators thereby triggering a fast forward response (Marino et al., 2014). Therefore, obviously a tight crosstalk exists, which goes into both directions, depending on the context. As a proof of principle, low sub-lethal levels of stress favor autophagy induction as a protective mechanism, whereas sustained stress beyond a certain threshold induces apoptosis. For instance, over-activation of autophagy can be a pro-death signal for autophagic cell death, and autophagy inducers can trigger apoptosis (Marino et al., 2014). Likewise, many stimuli that activate apoptosis can also stimulate autophagy. Extrinsic stress factors include chemotherapeutics, ionizing irradiation, lack of growth factors or nutrients. Intrinsically, p53 (see below), oncogenes (e.g., Myc), BH3-only proteins (Bcl-2 homology3) or serine/threonine kinases (such as JNK or AKT/PKB) are involved in the regulation of autophagy/apoptosis pathways (Marino et al., 2014). For instance, the pro-apoptotic Beclin1 and anti-apoptotic Bcl-2 are commonly affected. Both interact with each other and thereby regulate the balance between autophagy or apoptosis. Also, mitochondrial integrity, caspase and ATG activation, mTOR signaling and multiple others are implicated (Marino et al., 2014).

Collectively, a mis-regulation of autophagy in either direction is connected to numerous pathophysiological conditions, and the same holds true for apoptosis (Maiuri et al., 2007; Jiang and Mizushima, 2014; Marino et al., 2014).

#### Selective Autophagy

Specific cellular cargo can be selectively targeted by autophagy (Kirkin et al., 2009). The pathways have been named according to their type of cargo, for instance mitophagy for specialized autophagy of mitochondria (Ding and Yin, 2012; Hamacher-Brady and Brady, 2016; Khaminets et al., 2016), nucleophagy for removal of the nucleus (Park et al., 2009; Mijaljica and Devenish, 2013), ribophagy for ribosomes (Beau et al., 2008; Kraft and Peter, 2008; Frankel et al., 2017), and aggrephagy for protein aggregates (Yamamoto and Simonsen, 2011).

Mitophagy is fundamental for the mitochondrial homeostasis and a mis-regulation of mitophagy is clearly implicated in the development of neurodegeneration (see section “Distinct Neurodevelopmental Pathologies, Common Concepts – A Short Overview”). The key players of mitophagy, Parkin and PINK1 [tumor suppressor phosphatase and tensin homolog (PTEN)-induced putative kinase 1], are mutated in patients with Parkinson’s disease (Pickrell and Youle, 2015). Parkin functions as an E3-ubiquitin ligase, which is recruited to impaired mitochondria (Narendra et al., 2008). Parkin is required for ligation of ubiquitin marks to defective mitochondrial cargo (Ding and Yin, 2012; Harper et al., 2018). Parkin depends on the proper function of PINK1 (Vives-Bauza et al., 2010), an ubiquitin kinase located at the mitochondrial outer membrane (MOM) (Chin et al., 2010).

Mitophagy and apoptosis are both characterized by similar upstream events (Mukhopadhyay et al., 2014). Induction of mitophagy, for instance, is accompanied by activation of Bcl-2-associated X protein (BAX). This induces MOM perforation (MOMP), depolarization and release of cytochrome c from the mitochondrial intermembrane space (IMS) into the cytosol. As a consequence, PINK1 becomes stabilized at depolarized mitochondria and Parkin is subsequently translocated from the cytosol into the MOM (Vives-Bauza et al., 2010). By concerted action of both, mitochondria become decorated by ubiquitin marks (Lazarou et al., 2015) and can then be recognized by autophagy/mitophagy receptors such as the ubiquitin adaptor protein p62/sequestosome (Lamark et al., 2009; Geisler et al., 2010). In fact, autophagosome formation is mediated by different key mitophagy receptors such as optineurin and NPD52, which promote the recruitment of the autophagy initiating kinase ULK1 (Wong and Holzbaur, 2014; Heo et al., 2015; Lazarou et al., 2015). Nguyen et al. (2016) found that the GABARAP subfamily is essential for mitophagy. Meanwhile, the number of novel players involved in the complex process of selective autophagy is constantly expanding. Note that accumulation of damaged mitochondria, which are eliminated by mitophagy to a certain point, sets the threshold for apoptosis as a point of no return (Marino et al., 2014).

The autophagy receptor p62/sequestosome contains (i) interaction domains for ubiquitin, but at the same time also a LIR (LC3 interacting region) domain capable of binding to (ii) LC3, which itself is a key component of autophagosomal membranes. As a result, p62 recruits autophagosomal membranes to its selective, autophagosomal cargo (Lamark et al., 2009; Knaevelsrud and Simonsen, 2010).

**FIGURE 2:**
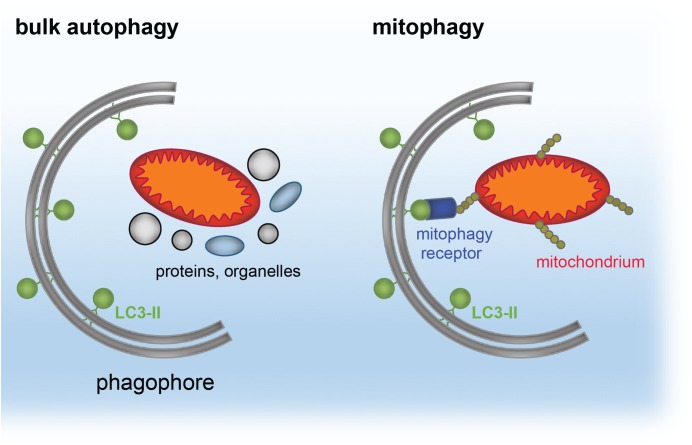
Simplified model of cargo targeted by bulk autophagy or mitophagy. The phagophore forms around bulk material, such as proteins and organelles during bulk (macro)autophagy. The phagophore is a double-membranous structure, which forms around the cargo and gives rise to the autophagosome. In contrast, mitophagy reflects selective recruitment of ubiquitinated (yellow) mitochondria (red/orange) by the mitophagy receptors (blue) to LC3-II (green) located at the phagophore.

**FIGURE 3:**
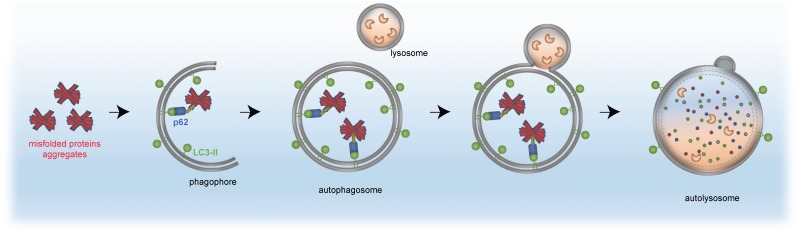
Removal of protein aggregates by selective autophagy. Aggregated proteins (red) are bound by the autophagy receptor p62 (blue), which itself has interaction domains for autophagosomal LC3 (green, lipidated LC3-II) and ubiquitin (yellow). The cargo is engulfed by the mature autophagosome and subsequently fuses with the lysosome to form the autolysosome, in which the cellular material is degraded by acidic hydrolases (orange).

The same principle of autophagy receptor (e.g., p62)/ubiquitin/LC3 cargo sequestration accounts also for removal of mis-folded protein aggregates, by a selective process termed aggrephagy (Lamark et al., 2009; Knaevelsrud and Simonsen, 2010; Yamamoto and Simonsen, 2011; Lamark and Johansen, 2012). Aggrephagy is also central to neurodegeneration (see section “Distinct Neurodevelopmental Pathologies, Common Concepts – A Short Overview”). A schematic for aggrephagy, bulk autophagy and mitophagy is depicted in Figure 2, 3.

### mTOR Signaling Couples Autophagy and Ribosome Biogenesis

The mammalian target of rapamycin (mTOR) pathway couples the intake of to nutrients, growth factors, energy and stress to the regulation of cell metabolism, growth, survival and autophagy (Pattingre et al., 2008). Deregulation is linked to various diseases and cancer formation.

Mammalian target of rapamycin signaling is recognized as essential pathway for proper neuronal development, neuronal survival and morphogenesis. Consequently, changes in mTOR signaling have been correlated with a spectrum of neuropathologies, such as epilepsy, intellectual disability, autism, brain injury, brain tumor formation and neurodegeneration (Crino, 2016; Switon et al., 2017). Likewise, mTOR inhibitors such as the bacterial macrolide rapamycin and its analogs are growingly used as therapeutic drugs and tested in clinical trials for effects in diverse neuropathological conditions (Laplante and Sabatini, 2009; Crino, 2016).

Mammalian target of rapamycin is a conserved serine-threonine kinase, which belongs to the phosphoinoside 3 kinase (PI3K) family. It assembles two large protein complexes, mTORC1 and mTORC2. The mTORC1 complex is considered as rapamycin sensitive complex (Pattingre et al., 2008). Rapamycin binds to FKBP12 and mTOR, thereby inhibiting mTORC1. mTORC1 signaling affects cell growth, metabolism and autophagy: Upon favorable conditions, mTOR is activated to allow cell growth by anabolic processes, such as rRNA biogenesis and protein translation. Upon nutrient deprivation and lack of growth factors, mTOR signaling is inhibited and cell growth is suppressed, whereas catabolic processes such as autophagy are induced to allow cell survival under unfavorable conditions. mTORC1 controls autophagy by regulating ULK1, ATG13 and FIP200, as well as by a reported rapamycin-insensitive mechanism (Laplante and Sabatini, 2009).

mTORC1 also regulates mitochondrial metabolism and biogenesis: mTORC1 inhibition impairs the MOM potential, reduces oxygen consumption and ATP levels. mTORC1 inhibition further decreases mitochondrial DNA levels and hampers mitochondrial biogenesis by affecting the transcriptional activity of the nuclear factor PGC1α (PPARγ co-activator 1) (Cunningham et al., 2007; Laplante and Sabatini, 2009).

Also p53 can regulate mTOR: DNA damage-induced p53 stabilization activates AMPK, which is a sensor of energy status and in turn results in mTORC1 inhibition. p53 also negatively controls mTORC1 by increasing *PTEN* expression, which functions as mTORC1 inhibitor. Inhibition of mTOR signaling diminishes nucleolar size and function and promotes longevity in different model organisms (Tiku and Antebi, 2018). However, the precise mechanisms regulating the crosstalk between ribosome biogenesis and autophagy remain to be determined.

**FIGURE 4:**
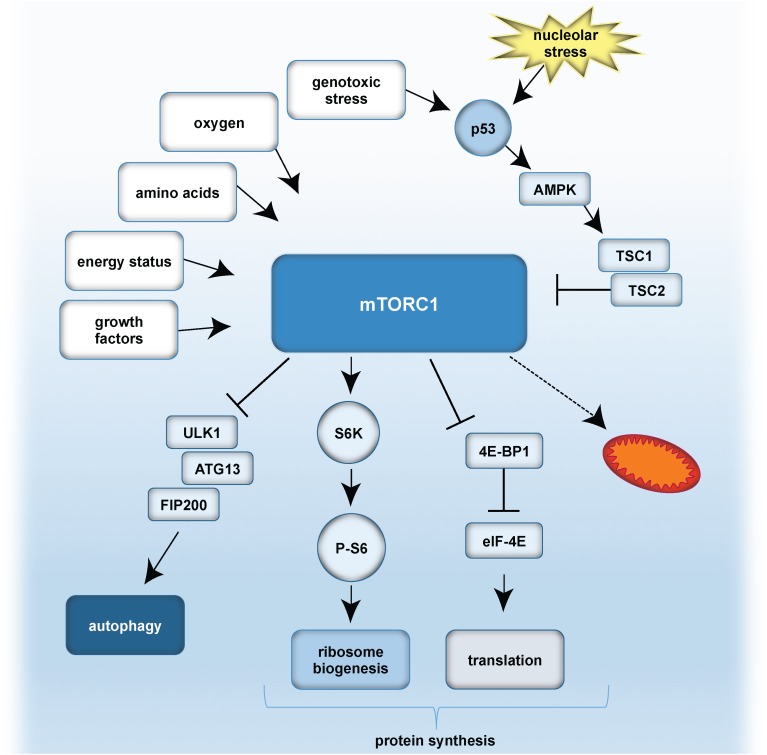
A simplified model of mTOR signaling, and effect of nucleolar stress on p53. Growth factors, energy status, amino acid availability, oxygen levels and genotoxic stress can result in mTORC1 activation. p53 is stabilized either by genotoxic stress and/or nucleolar stress. p53 inhibits mTORC1 by activation of AMPK and TSC1/TSC2 (Tuberous sclerosis proteins 1 and 2). mTORC1 further activates autophagy by inhibitory effects on the ULK1 complex, composed of ULK1, ATG13 and FIP200. mTORC1 promotes protein synthesis by (i) S6K activation, which stimulates phosphorylation of S6 and thereby ribosome biogenesis, as well as by (ii) inhibitory effects on 4E-BP1 and eIF-4E. As a consequence, translation is activated. Furthermore, mTORC1 influences mitochondrial biogenesis and metabolism.

A simplified model of mTORC1 signaling and the role of p53 is given in Figure 4.

## Nucleolar Stress and Autophagy: a Tight Regulation Between Health and Disease

Defective ribosome biogenesis on the one hand and impaired autophagy on the other hand are largely contributing to several diseases. In the following, an overview is provided on common concepts of three key classes of diseases, classically or recently connected to nucleolar stress and autophagy with specific focus on neurodegeneration, cancer and ribosomopathies. For a more detailed overview see for instance (Parlato and Kreiner, 2013; Ghavami et al., 2014; Danilova and Gazda, 2015; Woods et al., 2015; Hetman and Slomnicki, 2019).

### Distinct Neurodevelopmental Pathologies, Common Concepts – A Short Overview

The nervous system is vulnerable to intrinsic and extrinsic factors, which can give rise to distinct neurodevelopmental pathologies such as microcephaly, psychiatric disorders, autism, intellectual disability, epilepsy and neurodegeneration (please, be referred to review Hetman and Slomnicki, 2019). Causes include, for instance, gene mutations, infections or neurotoxins. As common concepts, gene expression, quality control mechanisms, cell proliferation, differentiation and apoptosis are mis-regulated. Apoptosis might give rise to microcephaly by eliminating, e.g., neuronal stem cells or post-mitotic neuronal cells (Hetman and Slomnicki, 2019). Likewise Zika virus infection, as an extrinsic factor for neurodevelopmental disorders, is tightly coupled to microcephaly. It has recently been demonstrated that it decreases mTOR signaling and over-activates autophagy (Liang et al., 2016). At the same time, ribosome biogenesis defects are emerging (reviewed in Hetman and Slomnicki, 2019).

Given the striking role of the nucleolus in coordinating mentioned neuropathological routes, deregulation of ribosome biogenesis rises as a potent upstream mechanism. In addition, also autophagy is activated in this context.

### Neurodegeneration and Aging

Aging represents a general risk factor for the formation of neurodegenerative diseases and consequently, neurodegeneration accumulates within our society. Despite the rapid advances made in medicine, not all negative aspects of aging can simultaneously be addressed. Along these lines, increasing the society’s age has to go together with improving anti-aging therapies. Our scientific knowledge on distinct neurodegenerative diseases has uncovered several common mechanisms, among them loss of neurons (Parlato and Kreiner, 2013; Parlato and Liss, 2014) and a prominent contribution of aggregate accumulation, induction of apoptosis and a mis-regulation of autophagy (Yamamoto and Simonsen, 2011; Ghavami et al., 2014). More recently, nucleolar stress has been connected to the induction of various types of neurodegenerative diseases, like Alzheimer’s, Parkinson’s, and Huntington’s Disease (see below) (Hetman and Pietrzak, 2012). In line, aging functions as susceptibility factor for neurodegeneration. It is characterized by a loss of rDNA and is accompanied by reduction of nucleolar size and a decline in rRNA processing (Garcia Moreno et al., 1997; Hetman and Pietrzak, 2012; Parlato and Kreiner, 2013). Therefore, the nucleolus is tightly connected to lifespan regulation (Tiku and Antebi, 2018).

As both routes of apoptosis and autophagy are interwoven and not yet fully understood, mechanistic research is essential as a basis for development of therapeutic approaches. As a mandatory goal, novel drugs have to be tested for specificity and efficacy.

#### Neurodegeneration – Underlying Concepts

In protein mis-folding diseases, also known as proteopathies, proteins loose their normal structure and/or function. As a result, mis-folded proteins accumulate and cause a toxic intracellular environment. Normally, proper cellular homeostasis is maintained by several machinery: the ubiquitin-proteasome system (UPS) degrades proteins, whereas autophagy is capable of removing proteins and whole organelles by the lysosome. Hence, both routes are essential for a healthy cell and both have been implicated in the development of neurodegenerative diseases (Levine and Kroemer, 2008; Ghavami et al., 2014). Note that increased apoptosis induction plays a crucial role in eliminating these damaged cells. In addition, chronic inflammation and oxidative stress are observed in neurodegenerative disorders.

Several neurodegenerative disorders, such as PD, AD, and HD are characterized by accumulation of mis-folded, ubiquitinated proteins, which damage the affected cell (Yamamoto and Simonsen, 2011). The accumulating proteins form inclusion bodies, which can differ between the distinct pathologies. The formation of inclusion bodies/aggresomes/amyloid structures represents a double-edged sword: aggregate formation is not only toxic, it can actually be considered as beneficial and neuroprotective mechanism by reducing the toxic nature of mis-folded ubiquitin-containing protein aggregates (Rubinsztein, 2006; Yang et al., 2007).

With increasing age the autophagic program looses efficiency, thereby increasing the likelihood of aggregate accumulation.

Neurons are highly sensitive to accumulation of protein aggregates and require proper autophagy mechanisms to keep the intracellular toxicity low. Supporting data demonstrating the significant implication of the autophagic machinery were, for instance, obtained in mice lacking ATG7 in the central nervous system. ATG-deficient mice, which fail to perform autophagy, display an accumulation of inclusion bodies followed by neuronal loss (Komatsu et al., 2006).

However, autophagy might have dual functions with respect to neurodegeneration: On the one hand functional autophagy is neuroprotective, by removing defective mitochondria via mitophagy. On the other hand pro-death autophagy is considered to induce neuronal cell death.

Massive inhibition of autophagy can trigger apoptosis, which is observed by loss of neurons in neurodegeneration. Interfering with autophagy regulators and blocking autophagy, results in accumulation of cargo-filled autophagosomes and lysosomes, again being toxic for the cell. As a consequence, lysosome-mediated cell death occurs (Kroemer and Jaattela, 2005). Impairment of mitophagy causes accumulation of defective mitochondria, which in turn induces reactive oxygen species (ROS) formation and mitochondrial apoptosis (Seo et al., 2010).

Induction of apoptosis by the chemotherapeutic agent staurosporine is accompanied by mitophagy and autophagy induction in dopaminergic cells. Additional block of autophagy by Bafilomycin or inhibition of mitophagy in PINK null mice sensitizes cells to staurosporine-induced apoptosis. Autophagy and mitophagy seems to be neuroprotective upon staurosporine-mediated apoptosis induction in dopaminergic neurons (Ha et al., 2014). However, with respect to loss of dopaminergic neurons, it is not fully resolved whether autophagy is beneficial or pathogenic.

Keeping mitochondria healthy is a prerequisite for counteracting neurodegenerative diseases. Concepts include for instance maintaining mitochondrial membrane integrity and functionality. Mitochondrial membrane permeabilization is tightly coupled to apoptosis induction (Kroemer et al., 2007). Also anti-oxidants and ROS scavenging appear as beneficial strategies. Inhibitors of apoptosis are used as therapeutic drugs to inhibit neuronal loss. Anti-apoptotic drugs prevent mitochondrial apoptosis by blocking release of cytochrome c from the mitochondria or activation of pro-apoptotic BAX (Westphal et al., 2014). Alternatively, the activity or abundance of anti-apoptotic factors can be elevated.

#### Alzheimer’s Disease

Alzheimer’s disease is the most common neurodegenerative disorder and has both sporadic and hereditary origin. Mostly, AD is diagnosed as sporadic form by the age of 65 years and represents the primary cause of dementia within the elderly generation (Seshadri et al., 1997; Ding et al., 2005).

Classically, prominent accumulation of defective mitochondria, increase of ROS, and apoptosis induction are found in patient’s neurons. A decline in autophagy during aging further promotes the mitochondrial release of cytochrome c, which serves as pro-apoptotic stimulus.

Also decreased nucleolar volume has been detected in AD patients (Iacono et al., 2008; Pietrzak et al., 2011). Mechanistically, inhibition of rDNA transcription by rDNA promoter methylation, reduced 28S/18S ratio, reduced tRNA abundance and increased rRNA oxidation has been linked to AD (Payao et al., 1998; da Silva et al., 2000; Ding et al., 2005; Pietrzak et al., 2011). In a similar manner, a decline of 28S rRNA was found in elderly healthy probes when compared to younger control groups (Payao et al., 1998; da Silva et al., 2000). The patient data suggest that impairment of ribosome biogenesis and protein synthesis is one of the earliest events observed in the pathogenesis of AD characterized by mild cognitive impairment (Ding et al., 2005).

#### Parkinson’s Disease

Parkinson’s disease is the second most common neurodegenerative disease after AD (Parlato and Liss, 2014). PD is characterized by loss of dopaminergic neurons in the brain stem. Patients display tremor, dementia and depression. Also in PD, most cases are sporadic. Typical risk factors are aging and exposure to (mitochondrial) toxins. A key feature of PD is deposition of Lewy bodies, which reflects deposition of α-Synuclein oligomers (Ghavami et al., 2014). These oligomers trigger mitochondrial damage. Hereditary forms of PD involve mutation of the key mitophagy regulators PINK and Parkin. Mutations of both result in impaired mitophagy. Given that α-Synuclein serves as a substrate of the E3-ubiquitin ligase Parkin, accumulation of α-Synuclein is also detected in a Parkin mutated background. Also ER stress is implicated in PD. However, mild ER stress is attributed to function rather in a neuroprotective manner by inducing pro-survival autophagy. PD is mimicked by treating neuronal cells with chemicals, such as MPP+ and rotenone, which trigger mitochondrial dysfunction and cell death (Nicklas et al., 1987).

More recently, disruption of nucleolar integrity has been observed in human post mortem samples of patients with PD (Rieker et al., 2011). In support of a nucleolar contribution, the ribosome biogenesis factor Nucleolin interacts with α-Synuclein. Consequently, damaged mitochondria, ROS, as well as autophagosomes accumulate and cause apoptosis (Rieker et al., 2011).

Disruption of nucleoli, cell cycle arrest and p53-mediated apoptosis is observed by depletion of the transcription initiation factor IA (TIF-1A), required for the recruitment of RNA pol I, in mouse embryonic fibroblasts (Yuan et al., 2005). Ablation of TIF-1A in DA neurons of mice results in Parkinsonism and progressive loss of DA neurons (Rieker et al., 2011) and likewise, reduced expression of *TIF-1A* is detected in PD patient samples (Evsyukov et al., 2017). Treatment with the neurotoxin MPTP worsens the effect of nucleolar stress. In this model, also p53 is stabilized and mTOR signaling decreased. Finally, ROS-mediated oxidative stress is induced and defects are detected in mitochondria, such as impaired mitochondrial transcription and COX (cytochrome c oxidase) activity (Rieker et al., 2011). Therefore nucleolar stress, by inhibiting mTOR signaling, can impair mitochondrial function, which represents a key hallmark of several neurodegenerative diseases.

To determine effects of specific PD mutations on nucleolar function irrespective of neuronal loss, pre-symptomatic, digenic PD models were analyzed. Mild overexpression of mutant human α-Synuclein in PINK1 null background (hA53T-SNCA/PINKKO) revealed differential nucleolar activity: On the one hand, reduced nucleolar activity and impaired nucleolar integrity was found in a subset of DA neurons, whereas others showed elevated nucleolar function, thereby suggesting possible compensatory mechanisms. In contrast, inactivation of PINK1 and DJ-1 showed no alterations, pointing to mutated α-Synuclein as the main contributor of nucleolar stress in the hA53T-SNCA/PINKKO model (Evsyukov et al., 2017).

Hemoglobin (Hb) is strongly expressed in dopaminergic neurons in the substantia nigra and is found in patient samples of AD and PD. Hb has recently been connected to mitochondrial function and apoptosis. Hb can form toxic aggregates in the nucleolus after stimulation with MPP+ and rotenone in a cellular model of PD. In turn, Hb overexpression impairs pre rRNA processing, induces nucleolar stress and sensitizes cells to apoptosis (Codrich et al., 2017). The authors further demonstrate decreased phosphorylation of the mTOR target 4E-BP1, decreased numbers of lysosomes in neurons and decreased levels of LC3-II following rotenone treatment, being indicative for inhibition of autophagy.

#### Huntington’s Disease

Huntington’s disease is caused by autosomal dominant mutation of the Huntingtin gene (*Htt*) and the onset of the disease is in average much earlier than AD and PD. Patients with HD display uncontrolled chorea movements and cognitive impairment (Ghavami et al., 2014). The onset of age also inversely correlates with the increasing number of repetitive Glutamine motifs present in mutated Huntingtin. Interestingly, capture of mutant Htt inside inclusion bodies was shown to be less toxic in comparison to accumulating free mutant Htt (Zuccato et al., 2010). Also in HD patients, apoptosis and mitochondrial damage is detected. Additionally, studies have demonstrated that rRNA transcription is affected in HD (Parlato and Kreiner, 2013). Triggering autophagy in mice models of HD can remove aggregates and increases their life span (Zheng et al., 2010).

Targeted disruption of nucleoli by conditional knockout of TIF-1A essential for the recruitment of RNA pol I in striatal neurons results in striatal degeneration and typical HD-like phenotypic alterations in mice (Kreiner et al., 2013). TIF-1A loss induces nucleolar disruption and nucleolar stress, which precedes neurodegeneration. Nucleophosmin (NPM) represents a multifunctional, nucleolar key factor involved in ribosome biogenesis, which fulfills a plethora of pro-survival processes (Colombo et al., 2011; Lindstrom, 2011). A down-regulation of NPM serves as readout for nucleolar stress induction and can be linked to neurodegeneration in several models of neurodegeneration (Marquez-Lona et al., 2012). In line, as a key marker for nucleolar stress (Colombo et al., 2002), NPM is reduced and p53 is stabilized in this model (Kreiner et al., 2013). As an early and p53-dependent pro-survival response, the p53 target PTEN (Stambolic et al., 2001) is induced in neurons. Given that the tumor suppressor PTEN counteracts the mTOR pathway, downstream targets of mTOR were analyzed for phosphorylation. It was found that p-S6 and p-4E-BP1 are reduced in the model. Inhibition of mTOR is connected to activation of autophagy and the same holds true in the HD model (Kreiner et al., 2013). Thus, transient over-activation of autophagy seems to be induced as initial, neuroprotective mechanism in response to impaired ribosome biogenesis. However, after sustained nucleolar stress, apoptosis of striatal neurons is inevitable (Kreiner et al., 2013; Parlato and Liss, 2014).

#### Cockayne Syndrome

DNA damage and impaired rRNA transcription are connected to Cockayne Syndrome (CS), which is a rare, congenital, autosomal-recessive neurodegenerative disorder (Karikkineth et al., 2017; Hetman and Slomnicki, 2019). Patients are characterized by premature aging, dwarfism, microcephaly, and have an average life expectancy of 12 years. Commonly mutated genes are CSA (20% of cases) and CSB (80% of cases), which are both, besides their key role in nucleotide excision repair, also required for RNA pol I transcription (Lebedev et al., 2008; Koch et al., 2014). CSB was found to localize to mitochondria and bind to mtDNA (Aamann et al., 2010; Kamenisch et al., 2010). CSB-deficient cells show increased ROS production, increased mitochondrial content and accumulation of damaged mitochondria, in line with impaired mitophagy (Scheibye-Knudsen et al., 2012). Further, CSB promotes acetylation of α-tubulin (Majora et al., 2018), which is a modification involved in cargo transport along microtubules to facilitate autophagosome/autolysosome fusion and aggresome clearance (Xie et al., 2010; Li et al., 2011). CSB deficiency abrogates autophagy and results in increased number of dilated lysosomes with impaired function (Majora et al., 2018). In human CS cells, translational infidelity is observed, most likely due to accumulation of error-prone ribosomes as a consequence of impaired ribosome replacement. CS cells exhibit ER stress and an over-activated unfolded protein response, which can be counteracted by addition of pharmacological chaperones (Alupei et al., 2018).

#### Epilepsy – A Disease Related to Neurodegeneration

Epilepsy is characterized by recurrent seizures and represents a disease related to neurodegeneration. Abrogated morphogenesis and synaptic function is observed upon nucleolar stress and could be connected to epilepsy (reviewed in Hetman and Slomnicki, 2019). For example, pharmacologically induced short-term seizures in mice transiently affect RNA Pol I activity in hippocampi and result in decreased *de novo* synthesized 18S and 28S rRNAs. In contrast, long-term seizures were associated with increased ribosome biogenesis (Vashishta et al., 2018). Epilepsy is further tightly linked to mTOR hyper-stimulation and autophagy over-activation (Cao et al., 2009; Zeng et al., 2009). Strikingly, administration of the mTOR inhibitor rapamycin counteracts seizures and thus functions in an anti-epileptogenic manner (Zeng et al., 2009).

#### Rare, Pediatric Neurodegenerative Diseases – A Short Outlook

Also rare, pediatric neurodegenerative diseases are characterized by alterations in autophagy. As an example, the multisystemic Vici syndrome is neurologically characterized by microcephaly and cognitive impairment. Accumulation of ubiquitinated autophagic cargo, p62 and damaged mitochondria is observed, reminiscent of neurodegeneration (Ebrahimi-Fakhari et al., 2014). However, whether also ribosome biogenesis is also altered here, remains to be determined.

### Cancer and Cancer Treatment

Key hallmarks of cancerous cells involve for instance mis-regulation of signaling pathways, rapid cell proliferation, accelerated tumor growth and inhibition of apoptosis (Hanahan and Weinberg, 2000, 2011). Large amounts of ribosomes are essential for cancerous cell growth and large nucleoli serve as prognostic marker in many tumor types (Montanaro et al., 2008; Penzo et al., 2019).

The nucleus arises as an essential target for cancer therapy (Woods et al., 2015; Pfister and Kuhl, 2018). Anti-tumor therapies utilize the high demand of cancer cells for the production of ribosomes by inhibiting RNA pol I. Inhibiting nucleolar structure/function and RNA pol I function has been characterized as beneficial in terms of triggering apoptotic cell death of cancer cells (Burger and Eick, 2013). Classical chemotherapeutics used in the clinics are for instance actinomycin D, 5-fluorouracil and metotrexat, which interfere with the nucleolar function (Boulon et al., 2010; Burger and Eick, 2013).

Novel drugs, which specifically impair rRNA transcription, are currently tested in clinical trials. The small molecule drug CX-5461 specifically inhibits transcription of RNA pol I and stabilizes p53, whereas RNA pol II is not affected. Also, translation and DNA replication is not impaired in human cancer cell lines (Drygin et al., 2011). The drug is further reported to impair proliferation in a p53-independent manner in cancer cell lines, whereas the effect on normal cell lines is minimal (Drygin et al., 2011).

Selective inhibition of RNA pol I by CX-5461 also robustly stimulates pro-death autophagy. Nucleolar stress and autophagy seem to be tightly coupled in distinct models and setups. Recently, CX-5461 was loaded on a nanoplatform to enrich for nucleolar accumulation of the drug in order to enhance the anti-cancer effect, without causing significant side effects. *In vivo* and *in vitro* assays confirm induction of pro-death autophagy in HeLa cells, as well anti-proliferative and anti-tumor effects (Duo et al., 2018).

Besides autophagy, CX-5461 induces also senescence in a p53-independent manner. In U2OS osteosarcoma cells, CX-5461 induces G2 arrest, but not apoptosis (Li et al., 2016). In response to CX-5461, p53 accumulates and *p21* is induced. In addition, increased levels of LC3-II are detected under basal conditions. Using TEM analysis, the authors noticed expanded vacuole-like structures filled with organelles, however, they report lack of clear identification of autophagosomal character. Knockdown of p53 by siRNA rescues p21 up-regulation and LC3-II accumulation and increases cell survival. The authors conclude that CX-5461 triggers p53-dependent autophagy. Autophagy occurs via the AMPK/mTOR pathway in U2OS cells, as evidenced by reduced p-mTOR and increased p-AMPK levels (Li et al., 2016). The p53 target p21 is shown to be up-regulated during autophagy and a p53-independent increase of p21 is reported in MNNG cells with mutant p53 (Li et al., 2016).

Taken together, autophagy induction as a response to nucleolar stress seems to be an initial surveillance mechanism in several models. However, also in terms of cancer, autophagy induction can have two modes of action: Autophagy induction is clearly beneficial for cells by preventing genotoxic stress and DNA damage. It removes cellular sources of ROS, such as defective mitochondria or proteins (Mrakovcic and Frohlich, 2018). In contrast, inhibition of autophagy represents an oncogenic event. At later stages over-activation of autophagy facilitates oncogenic drug-resistance. Autophagy inhibitors chloroquine and hydroxychloroquine have therefore been tested in clinical trials for cancer therapy (Yang et al., 2011; Marino et al., 2014).

### Ribosomopathies

Impairment of ribosome biogenesis is connected to a diverse class of human diseases collectively termed ribosomopathies (Freed et al., 2010; Narla and Ebert, 2010; Danilova and Gazda, 2015; Yelick and Trainor, 2015). Patients exhibit either mutations and/or haploinsufficiency of RPs or ribosome biogenesis factors. Several players associated with ribosomopathies have been described (see also below). Classically, the nucleolar stress response and the tumor suppressor p53 are activated (Freed et al., 2010; James et al., 2014). Despite a common mechanism of interfering with ribosome biogenesis, the patient’s phenotypes differ among the distinct syndromes. Intriguingly, though, some phenotypes are common and include defects of the craniofacial cartilage, anemia and increased cancer susceptibility. The elevated cancer risk appears paradoxical, given the great need of tumor cells for large amounts of ribosomes (Montanaro et al., 2008). Accordingly, pathways and mechanisms might well exist, which let both co-exist (Pfister and Kuhl, 2018). For example, specialized onco-ribosomes have recently been uncovered to increase the cellular fitness by mediating preferential translation of anti-apoptotic Bcl-2, as observed for the ribosome mutant RPL10-R98S in leukemia cells (Xue and Barna, 2012; Sulima et al., 2017; Kampen et al., 2018). However, the question on cause and consequence of ribosomopathy-induced cancer formation is still under debate.

Recently, examination of murine hepatocellular carcinoma and hepatoblastoma has revealed ribosomopathy-like features of nucleolar stress, such as deregulated expression of RPs and accumulation of unprocessed rRNA precursors. Despite the fact that p53 is stabilized, no growth inhibition occurs (Kulkarni et al., 2017). Therefore, compensating mechanisms might counteract apoptosis, involving up-regulation of anti-apoptotic Bcl-2, silencing of p19 ARF or cytosolic sequestration of p53. Those events would in turn inhibit the tumor suppressive mechanisms of cell cycle arrest and apoptosis in these cancers (Kulkarni et al., 2017).

Clearly, ribosome biogenesis is a highly energy-consuming process. An implication of autophagy comes in mind, which might compensate for the low levels of functional ribosomes observed in ribosomopathies. Many important questions arise. Is there a connection to bulk autophagy or selective autophagy of ribosomes (ribophagy) in ribosomopathy patients? Until now broad studies are lacking, which precisely address their implication in these issues. However, first data are collected, which indeed unravel involvement of autophagy in these processes (see section “Nucleolar Factors and Nucleolar Stress in the Regulation of Autophagy and *Vice Versa*”).

An overview summarizing the emerging connection between nucleolar stress and autophagy in the diseases presented here is given in Table 1.

**Table 1:** The role of nucleolar stress in mentioned diseases and effects on autophagy.

Condition/Disease^1^	Effects related to nucleolar stress^1^	Effects related to autophagy^1^
Aging	Loss of rDNA rRNA processing impaired Nucleolar size reduced	Autophagy impaired
Alzheimer’s Disease	rDNA transcription impaired Nucleolar volume decreased	Autophagy impaired Defective mitochondria
Parkinson’s Disease	Nucleolar disruption Altered nucleolar function	mTOR pathway inhibition Autophagy altered Mitophagy impaired Defective mitochondria
Huntington’s Disease	rDNA transcription impaired NPM reduced	mTOR pathway inhibition Autophagy transiently over-active Defective mitochondria
Cockayne syndrome	rDNA transcription impaired	Autophagy impaired Defective mitochondria
Epilepsy	RNA pol I altered	mTOR pathway activation Autophagy over-active
Ribosomopathies	rRNA processing impaired mutated RPs	mTOR over-activation Autophagy over-active ROS
Cancer	Need for ribosomes Large nucleoli Ribosomopathy-like	Autophagy conveys drug resistance Autophagy has dual roles
Zika virus infection	Nucleolar NPM displacement	mTOR pathway inhibition Autophagy over-active

*^1^Please, see text for references and details.*

## Nucleolar Factors and Nucleolar Stress in the Regulation of Autophagy and *Vice Versa*

Recently, inhibition of RNA pol I has been connected to autophagy, revealing that nucleolar stress functions upstream of autophagy. In the following, evidence is collected, which links the ribosome biogenesis machinery and the nucleolus to autophagy, and *vice versa*. As a common principle, different groups suggest implication of mTOR signaling in nucleolus-mediated autophagy (see below). Also here, p53-dependent and -independent pathways are being identified.

### The p53 Family

Besides the classical role of p53 as guardian of the genome, by mediating cell cycle arrest and apoptosis, p53 has been reported to exert distinct roles in autophagy (Wang et al., 2014). This depends on its subcellular localization: nuclear, cytosolic or mitochondrial, respectively.

The effect of nuclear p53 as an inducer of autophagy mostly depends on its role as transcription factor. Nuclear p53 induces expression of ATGs thereby driving autophagy. In line, many promoters of autophagy related factors, such as ATGs or Parkin, are occupied by p53 (Zhang et al., 2011; Fullgrabe et al., 2016). Induction of pro-apoptotic target genes implicated in mTOR activation, such as *TCS2*, *AMPK*/*PTEN* and *Sestrin*, result in autophagy activation. The p53 target *DRAM* is directly involved in autophagosome formation. P53 further induces *BAX* and *Bcl-2* or *DAPK*, which in turn induce *Beclin1* (Mrakovcic and Frohlich, 2018). Moreover, the p53 family members p63 and p73 induce expression of the autophagy regulators *ATG5* and *ATG7*. Also E2F, an important co-regulator of p53, is involved in the transcriptional regulation of autophagy related genes (Polager and Ginsberg, 2009; Fullgrabe et al., 2016). Note that other p53 family members p63 and p73 can, in principal, compensate for a loss of p53 (Kenzelmann Broz et al., 2013; Fullgrabe et al., 2016). However, it remains to be determined, whether p53-independent pathways actually depend on the role of p63 and p73 or whether they are unrelated.

Cytosolic p53 counteracts autophagy by transcription-independent mechanisms. P53 inhibits AMPK and activates mTOR, p53 further interacts with Beclin and induces Beclin ubiquitination and degradation (Mrakovcic and Frohlich, 2018). Cytosolic p53 interacts with Parkin, which is the key regulator of mitophagy. It was reported that p53 counteracts Parkin recruitment to mitochondria, thereby impairing mitophagy (Hoshino et al., 2013).

Mitochondrial p53 has a plethora of functions: it triggers MOM permeabilization, ROS production, mitophagy and autophagy and is therefore implicated in neuropathological conditions (Marino et al., 2014; Wang et al., 2014).

### mTOR Inhibition by Rapamycin

mTORC1 inhibition by rapamycin abrogates the nucleolar stress response induced by low, cytostatic doses of the chemotherapeutic actinomycin D (Goudarzi et al., 2014). As a result, p53 stabilization and p21 induction is impaired. The authors observe decreased interaction of RPL11 with MDM2 upon rapamycin and actinomycin D co-treatment and suggest a mechanism related to the classical RPL11-MDM2-p53-pathway. Also, they detected decreased RPL11 levels, as well as MDM2 stabilization, which might in part contribute to the rapamycin-mediated effects on p53. Interestingly, inhibition of mTOR by caffeine at physiologically relevant doses is capable of abrogating the actinomycin D-induced p53 response (Goudarzi et al., 2014). Thus, a complex network between mTOR inhibitors/autophagy, nucleolar stress and cancer treatment is established.

### RNA pol I Inhibition and NPM

RNA pol I inhibition by actinomycin D or adriamycin is well known to trigger nucleolar disruption. Recently, the observation has been made that also autophagy can be induced with these drugs (Katagiri et al., 2015). The same holds true for knocking down the RNA pol I transcription factors TIF1A and POLR1A. Induction of autophagy has been monitored in flux experiments by counting LC3 punctae (being indicative for autophagosomal number) and determining levels of lipidated LC3-II (representing the autophagosome-bound form of LC3). At the same time the autophagy substrate p62/sequestosome is reduced, indicating increased turnover by autophagy. Autophagy induction can be rescued by treating cells with autophagy inhibitors or knocking down key autophagy regulators ATG5 and Beclin1. In contrast, nucleolar disruption is not rescued (Katagiri et al., 2015). Together, this finding places nucleolar disruption upstream of autophagy. As nucleolar stress is generally characterized by redistribution of nucleolar factors (such as NPM), or p53 stabilization, Katagiri et al. (2015) have determined, which accounts for the effects observed. They found that induction of autophagy by TIF1A knockdown is independent of p53, but depends on NPM. The induction of autophagy can be rescued by NPM knockdown, without reducing p53 levels. In contrast, neither the depletion of NPM affects starvation-induced autophagy; nor does nutrient deprivation have an impact on nucleolar integrity. This suggests that NPM might rather play a role in a specialized form of nucleolar stress-induced autophagy, than starvation-induced bulk autophagy.

### The Nucleolar Factor – PICT-1

The nucleolar factor PICT-1/GLTSCR2 is considered a tumor suppressor, as it binds and stabilizes PTEN. In contrast, PICT-1 deletion is linked to cancer formation and functions as oncogenic regulator of the E3-ubiquitin ligase MDM2 by preventing nucleolar release of RPL11 (Sasaki et al., 2011; Suzuki et al., 2012). Consequently, p53 stabilization, G1 cell cycle arrest and apoptosis are observed, thereby counteracting tumor growth (Sasaki et al., 2011). Homozygous PICT1 deletion in mice is lethal and impairs survival of mouse ES cells. PICT-1 binds to rDNA and the RNA pol I transcription factor upstream binding factor-1 (UBF-1). It inhibits transcription of rRNA, which depends on its localization to nucleoli (Chen et al., 2016). With respect to autophagy, it has been shown that PICT-1 overexpression induces GFP-LC3 punctae and reduces p62 levels, and that it inhibits the AKT/mTOR/p70S6K pathway (Chen et al., 2016). As the autophagy inhibitor 3-MA (3-methyladenine) enhances cell death upon PICT-1 overexpression, the authors suggest induction of pro-death autophagy. Together, the authors conclude that PICT-1 overexpression triggers pro-death autophagy, without inducing the classical nucleolar stress response, such as p53 stabilization and nucleolar disruption (Chen et al., 2016).

### The Ribosomopathy Factor – SBDS

Recent findings suggest that autophagy might be affected in patients with ribosomopathies. mTOR signaling regulates a variety of essential cellular processes, among them autophagy. In leukocytes derived from patients with the ribosomopathy syndrome Shwachman Bodian Diamond Syndrome (SBDS), a hyper-activation of mTOR phosphorylation is observed (Bezzerri et al., 2016). Also in intestinal epithelial cells autophagy is over-activated. In this context, autophagy is independent of mTOR or p53 and is induced as a consequence of nucleolar/ribosomal stress (Bezzerri et al., 2016).

### The Ribosomopathy Factor – RPS19

Disrupted ribosome biogenesis by knocking down RPS19 (ribosomal protein S19) is connected to the ribosomopathy syndrome Diamond Blackfan Anemia (DBA). RPS19 knockdown further affects autophagy in patient cells, and autophagy induction is also observed in red blood cells of zebrafish embryos (Heijnen et al., 2014).

RP-deficiency also recapitulates these effects in cells derived from SBDS. An increase in P-S6 as well as ROS is observed, whereas anti-oxidant treatment reverses p-S6, autophagy and p53 stabilization (Heijnen et al., 2014). Thus, the observed effects turn out to be ROS-dependent and suggest a contribution of oxidative stress in ribosomopathies.

### The Ribosomopathy Factor – pwp2h

The zebrafish titania mutant (tti^s450^) harbors a recessive, lethal mutation of the *pwp2h* gene encoding a factor of the small ribosomal subunit (Boglev et al., 2013). In this mutants reduced 18S rRNA, impaired ribosome biogenesis and p53 stabilization is observed. *pwp2h* is highly expressed in intestinal, epithelial cells, but also in the brain retinal pigmented epithelium, liver and pancreas, which are rapidly dividing tissues. Also here, defects in craniofacial formation, typical hallmarks of ribosomopathies, can be detected.

Intestinal epithelial cells of the mutant larvae display accumulation of autophagosomes. In autophagic flux experiments using the autophagy inhibitor chloroquine and activator rapamycin, increased accumulation of LC3-II is observed in the mutants, indicating autophagy induction (Boglev et al., 2013). Also p-RP-S6, reflecting mTORC1 activity, is increased. Inhibiting autophagy by morpholino-mediated knockdown of ATG5 triggers apoptosis of intestinal epithelial cells specifically in the mutants, whereas the wildtype is not affected. This suggests that autophagy induction counteracts apoptosis as survival mechanism in response to nucleolar stress. Also, no signs of apoptosis are detected in the mutants, ruling out toxic levels of autophagy activation. Interestingly, autophagy induction in the zebrafish mutants occurs in mTOR and p53-independent manner (Boglev et al., 2013). However, the molecular mechanisms and pathways affected remain elusive.

### The *Drosophila* Nopp140

Nopp140 is in structure and function related to Treacle, representing an essential gene in the ribosomopathy syndrome Treacher Collins syndrome (TCS) (Valdez et al., 2004; Sakai and Trainor, 2009; Dai et al., 2016). Depletion of the nucleolar phosphoprotein Nopp140 in the imaginal wing disks of *Drosophila* results in nucleolar stress, loss of ribosomes and p53-independent apoptosis (James et al., 2013). Since there are no detectible levels of MDM2, NPM/B23 and ARF in *Drosophila*, the authors conclude that an alternative nucleolar stress response might exist. They consider JNK as an interesting link, which has earlier been shown to induce autophagy in response to oxidative stress and induce transcription of *ATG* genes (Wu et al., 2009; Zhou et al., 2015). In addition, oxidative stress induces accumulation of the autophagy marker GFP-LC3 and lysosomes in the intestinal epithelium, which is dependent on JNK signaling (Wu et al., 2009). As also in larval polyploidy midgut cells mCherry-ATG8a is accumulated after Nopp140 depletion, the authors conclude an accumulation of autophagosomes and a premature induction of autophagy regulated by loss of Nopp140 (James et al., 2013).

### NAT10 and Glucose Deprivation Stress

NAT10 drives ribosome biogenesis by mediating acetylation of the RNA pol I transcription factor UBF-1 and facilitating processing of the 18S rRNA (Kong et al., 2011; Ito et al., 2014). Under normal conditions, NAT10 is auto-acetylates and promotes recruitment of PAF53 and RNA pol I to mediate rRNA biogenesis (Cai et al., 2017), whereas autophagy is inhibited (Liu et al., 2018).

The mechanisms, which link inhibition of rRNA biogenesis to induction of autophagy in response to energy stress were determined by Liu et al. (2018). They demonstrate that NAT10 binds and acetylates the autophagy regulator Che1 (AATF) in a p53-independent manner. As a consequence of acetylation, the transcriptional activation of target genes *Redd1* and *Deptor* is off. Thus, Che1 enhances autophagy by activating the transcription of *Redd1* and *Deptor*, two critical inhibitors of mTOR signaling (Desantis et al., 2015a).

Interestingly, Che-1 is also important for RNA pol II, the DNA damage response (DDR) and drives p53 expression. Upon DDR, Che1 increasingly interacts with p53 and drives the expression of genes implicated in cell cycle regulation, for instance *p21* (Desantis et al., 2015b).

Upon energy stress and glucose deprivation, Sirt1 deacetylates NAT10. ChIP analysis has demonstrated that deacetylated NAT10 does not bind to rDNA upon glucose deprivation and thus NAT10-mediated ribosome biogenesis is inhibited. Under this condition, the inhibition of Che1 is released (Liu et al., 2018).

In HCT116 cells, LC3-II levels are increased both in presence or absence of chloroquine, showing that NAT10 knockdown increases basal autophagic flux. Also, p62 is reduced upon NAT10 depletion. Strikingly, the effects observed are independent of p53, as demonstrated in HCT116 p53^−/−^ cells. In rescue experiments with HCT116-NAT10-Cas9 knockout clones, overexpression of NAT10 reverses the effects observed on p62 and LC3-II, whereas acetylation mutants fail to do so (Liu et al., 2018). Glucose withdrawal triggers release of NAT10 from nucleoli. Treatment of cells with RNAseA also leads to a loss of NAT10 from nucleoli and impairs binding to UBF-1, suggesting that rRNA binding is essential for nucleolar accumulation of NAT10. In contrast, the acetylation status does not matter as determined by use of a NAT10 mutant.

Together, Sirt1 mediated deacetylation of NAT10 impairs rRNA biogenesis and results in release of NAT10 from nucleoli. Therefore, both regulate the switch between ribosome biogenesis and autophagy as a response to energy stress to maintain cellular survival.

### The Autophagy Regulator LC3/Atg8 – Present in the Nucleolus

Besides its localization in the autophagosomal membranes and the cytosol, LC3 can rapidly shuttle into and out of the nucleus. Endogenous and overexpressed LC3 has been found to be associated with nucleoli. However, the authors point out a weak signal being indicative for a low degree of enrichment (Kraft et al., 2016). A triple arginine motif is essential for the nucleolar targeting of LC3. The arginine motif mediates protein-protein interaction and binding to RNAs, suggesting accumulation at nucleoli by interaction with rRNAs and/or nucleolar RNA binding proteins (Zhou et al., 1997; Behrends et al., 2010). Likewise, LC3 has been connected to interaction with the 60S ribosomal subunit (Kraft et al., 2016). In addition, a hydrophobic binding interface contributes to nucleolar localization. In contrast, the lipidation site of LC3 is dispensable for nucleolar targeting. Interestingly, several 40S RPs such as S27, S5, S18, and S20, have been identified as interaction partners of LC3 by MS analysis. The authors speculate that nucleolar LC3 might counteract p53 stabilization by preventing interaction of RPs with MDM2 (Kraft et al., 2016).

### TP53INP2/DOR

TP53INP2/DOR (tumor protein p53 inducible protein nuclear protein 2) is a nuclear protein, which interacts with the LC3-related GABARAP, GABARAP-like2, as well as with LC3 via its N-terminus. TP53INP2/DOR also binds to VMP1, a transmembrane protein of autophagosomes. Upon starvation by rapamycin or EBSS incubation, TP53INP2-EGFP translocates from nuclei to the cytoplasm where it co-localizes with LC3 family proteins, indicating autophagosomal recruitment (Nowak et al., 2009). Knockdown of TP53INP2 in HeLa cells reduces rapamycin-induced accumulation of LC3-II, as well as the number of RFP-LC3 punctae per cell. Also, less Beclin1 is recruited to the autophagosomes upon knockdown of TP53INP2. The rapamycin-induced recruitment of TP53INP2 to autophagosomes is dependent of autophagy: it depends on ATG5, as demonstrated in ATG5^−/−^ MEFs, and it is stimulated by rapamycin. Further, it depends on PI3K as revealed by inhibition by the PI3K inhibitor wortmannin (Nowak et al., 2009).

Huang and Liu (2015) determined the molecular mechanisms underlying recruitment of LC3 from the nucleus to the cytoplasm (Liu and Klionsky, 2015). By use of an NES mutant of TP53INP2/DOR, which cannot exit the nucleus, LC3 is captured in the nucleus under starvation. This data suggest that TP53INP2/DOR mediates export of nuclear LC3 during autophagy (Huang and Liu, 2015). Also, loss of Beclin1 and ATG14 inhibits the exit (Huang and Liu, 2015). Upon nutrient deprivation, SIRT1 deacetylates nuclear LC3 at K49 and K51. SIRT1 is activated by metabolic stress and functions as essential activator of autophagy by deacetylating its substrates, among them p53 and ATGs (Vaziri et al., 2001; Lee et al., 2008; Lapierre et al., 2015). This increases the interaction of TP53INP2/DOR with deacetylated LC3 and mediates cytosolic export of LC3. The authors show that LC3 derived from the nucleus is the primary source of membrane-bound LC3. As a consequence, LC3 is lipidated and autophagosome biogenesis is initiated. Therefore, TP53INP2/DOR has a key role as a scaffold for LC3, by mediating LC3 lipidation. SIRT1 is essential for deacetylation, which is required for the interaction with ATG7 (Huang and Liu, 2015).

The autophagy regulator TP53INP2/DOR has recently been found in nucleoli. TP53INP2/DOR-RFP co-localizes with Nucleophosmin-GFP and Fibrillarin in HeLa cells. Upon co-expression of a dominant negative NPM mutant, the double leucine mutant NPMdL, which cannot exit the nucleus (Maggi et al., 2008), TP53INP2/DOR is still able to perform nucleo-cytoplasmic shuttling. However, on longer term its localization to the nucleolus is impaired (Mauvezin et al., 2012). Localization of TP53INP2/DOR is mediated by a C-terminal nucleolar localization signal (NoLS) (Xu et al., 2016). ChIP analysis identified binding to rDNA, and a nucleolar exclusion of TP53INP2/DOR led to impaired rRNA synthesis. TP53INP2/DOR is capable of directly binding to the RNA pol I pre initiation complex.

In summary, TP53INP2/DOR has a dual function by mediating ribosome biogenesis in the nucleolus and regulating autophagy.

### *Drosophila* – RPD3

Rpd3 represents a *Drosophila* homologue of histone deacetylase 1 (HDAC1). At early stages of starvation, Rpd3 accumulates in the nucleolus and co-localizes with the nucleolar factor Fibrillarin in *Drosophila*. Following starvation, 18S rRNA and Rpd3 mRNAs increases transiently. ChIP analysis has demonstrated that Rpd3 binds to the rDNA promoter. Rpd3 upregulates rRNA synthesis, whereas a Rpd3 knockdown reduces the number of polysomes during starvation (Nakajima et al., 2016). Also, knockdown flies die faster in response to starvation. In the knockdown flies, reduced levels of *Atg9* mRNA are detected, which is induced in response to starvation in controls, revealing decreased tolerance to starvation-mediated stress.

### *Drosophila* – Nucleostemin-Like 2

In *Drosophila*, nucleostemin-like protein NS2, which is a homologue of human NGP1/GNL2, localizes to nucleoli. Loss of NS2 results in ribosomal stress and block of nucleolar release of 60S subunits as evidenced by increased GFP-RPL11 in nucleoli (Wang and DiMario, 2017). In polyploid midgut cells, mCherry-Atg8a positive autophagosomes are detected by immunofluorescence analysis, as well as autophagosomes containing mitochondria by TEM analysis. In contrast, in larval imaginal disks induction of apoptosis is observed (Wang and DiMario, 2017).

### CLIP – Nucleophagy of Nucleolar Factors in Yeast

In budding yeast, nutrient deprivation and TORC inhibition triggers nucleophagy, the selective degradation of the nuclear compartment. In particular, nucleolar factors are degraded,whereas rDNA is excluded (Mostofa et al., 2018). The authors establish that autophagy induction by rapamycin triggers the redistribution of nucleolar proteins and rDNA, thereby separating rDNA from nucleophagy. CLIP and cohibin, which are essential for tethering rDNA to the inner nuclear membrane, are responsible for the repositioning of rDNA and nucleolar proteins in yeast. They are also required for the nucleophagic degradation of nucleolar factors. In contrast, rDNA is not degraded by macro-nucleophagy (Mostofa et al., 2018).

Thus, starvation-mediated autophagy, at least in yeast, removes specifically nucleolar factors. It seems likely that autophagy allows inhibition of the energy-consuming process of ribosome biogenesis by selectively removing the processing machinery.

**FIGURE 5:**
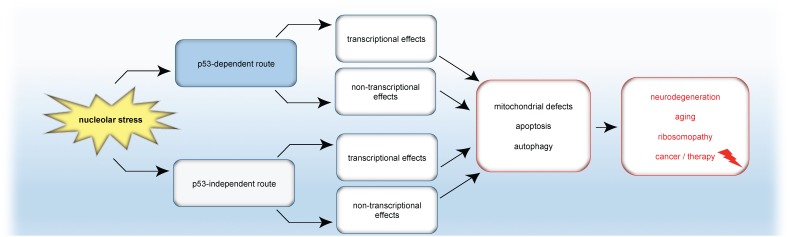
Model: Nucleolar stress in apoptosis, autophagy and disease. Nucleolar stress triggers either the classical p53 response or p53-independent mechanisms. In turn, transcriptional programs and/or transcription-independent mechanisms are induced, which finally cause mitochondrial changes, autophagy or apoptosis. As a consequence of nucleolar stress, not only apoptosis but also autophagy is emerging as a tightly coupled stress response pathway for the formation or cure of pathological conditions.

A simplified model summarizing the emerging connection between nucleolar stress and autophagy presented in this review is given in Figure 5.

## Closing Remarks and Perspectives

Despite the recent advances made in uncovering the relationship between nucleolar stress and autophagy, our understanding is far from being complete. A critical need to elucidate the underlying causes is apparent.

On a mechanistic level it seems likely that p53-dependent as well as -independent effects account for the induction of the autophagic nucleolar stress response.

It might well be that reduced ribosome biogenesis, by reducing protein synthesis, triggers autophagy as a general stress response. Nevertheless, extra ribosomal functions might as well coincide.

As precise underlying mechanisms are currently missing, many questions and possibilities rise: How are both signaling pathways integrated with each other? Which effects are cell type and context dependent? Under which conditions is autophagy either beneficial or a contributor to the pathology?

Is there a p53 or compensatory p63/p73 autophagy response triggered in nucleolar stress? Does selective autophagy and other forms of autophagy such as micro-autophagy play a fundamental pathogenic role in other diseases connected to nucleolar stress?

The initial and common concepts of nucleolar stress and autophagy open novel avenues for investigating specific therapeutic approaches. Many autophagy inhibitors and activators might be contemplated as therapies for nucleolar-stress mediated diseases. They might well be combined with, e.g., RNA pol I inhibitors and tested for synergy to increase selectivity but simultaneously reduce toxicity for patients.

To get a deeper understanding of the underlying events, a challenge will be to elaborate state of the art autophagy methods on monitoring the autophagy flux in cellular models of diseases, patient tissues and blood samples (Jiang and Mizushima, 2014). Research on the autophagic transcriptome and spatio-temporal expression patterns of regulators of the autophagic machinery or nucleolar response will advance our knowledge on mechanisms coupling the nucleolar stress response to autophagy.

## Author Contributions

AP performed the literature research, conceptualization, and wrote the review.

## Conflict of Interest Statement

The author declares that the research was conducted in the absence of any commercial or financial relationships that could be construed as a potential conflict of interest.
